# The coronary sinus reducer for the treatment of refractory angina

**DOI:** 10.1007/s12471-016-0903-x

**Published:** 2016-10-26

**Authors:** Y. Paz, A. Shinfeld

**Affiliations:** 1 General Intensive Care Unit, Tel Aviv Sourasky Medical Center, affiliated with Sackler Faculty of Medicine, Tel Aviv University, Tel Aviv, Israel; 2 Department of Cardiac Surgery, Chaim Sheba Medical Center, Tel Hashomer, affiliated with Sackler Faculty of Medicine, Tel Aviv University, Tel Aviv, Israel

To the Editor,

We were very interested to read the recently published manuscript ‘Safety and efficacy of a device to narrow the coronary sinus for the treatment of refractory angina: A single-centre real-world experience’ by Masieh Abawi et al. [1]. Some people make a mistake and compare the coronary sinus reducer (CSR) with the Beck procedure (Beck operation), but actually the Beck procedure has almost nothing in common with the CSR.

In the 1940s, Dr Claude Beck described two types of coronary sinus (CS) interventions [2, 3]. The Beck I procedure consisted of the following steps: 1) abrading of both epicardium and inner pericardium. Dr Beck explained that it produces mechanical trauma, which in turn causes inflammation and intercoronary arterial channels. 2) spilling of powdered asbestos and 5 % aqueous trichloracetic acid on the epicardium. Dr Beck explained that this produces chemical trauma, which in turn also results in inflammation and intercoronary arterial channels. 3) CS constriction to a diameter of 3 mm. The Beck II procedure consisted of a vascular graft between the descending aorta and the CS followed by operative external constriction of the CS ostium a few weeks later. Both procedures needed thoracotomy and as can be seen have very little in common with the CSR.

May we add our personal experience with the CSR: In the mid-1990s we tested a new strategy supporting the ischaemic myocardium. This strategy included: 1) catheterisation of the coronary venous system rather than catheterisation of the coronary arteries, and 2) reduction of the CS effective cross area, as opposed to expansion of a narrowed coronary artery. At that time, the main concept behind this strategy was only to rebuild retrograde coronary pressure that would be attenuated by the atherosclerotic disease. In order to test this strategy, we designed and manufactured the first CSR stent. In a preliminary non-ischaemic pig model we succeeded in increasing the mean CS pressure from 7.0 to 24.6 mm Hg (*p* = 0.011) immediately after CSR stent deployment. Further studies in a non-ischaemic pig model were devoted to macroscopic and histological investigations of the treated hearts, in particular investigating whether any structural or histological damage, such as an infarct, had occurred after reducer implantation. While looking for such damage, these studies revealed that 8 to 12 weeks of CS narrowing produced macroscopic epicardial and intramyocardial new blood vessels – neovascularisation. Histopathological analysis described these findings as follows: significant proliferation of small to medium-sized vessels, containing smooth muscle representing coronary collaterals. This was evident in almost all specimens, representing various myocardial anatomical areas, including specimens from the anterior and mid-posterior wall. According to these unpredicted neovascularisation findings after reducer implantation in a non-ischaemic pig model, we created the name ‘Neovasc’ for this novel CSR stent.
